# A Role for the Brain RAS in Alzheimer’s and Parkinson’s Diseases

**DOI:** 10.3389/fendo.2013.00158

**Published:** 2013-10-25

**Authors:** John W. Wright, Leen H. Kawas, Joseph W. Harding

**Affiliations:** ^1^Departments of Psychology, Integrative Physiology and Neuroscience, Program in Biotechnology, Washington State University, Pullman, WA, USA

**Keywords:** angiotensin II, angiotensin IV, hepatocyte growth factor, angiotensin receptors, c-Met receptor, Mas receptor, Alzheimer’s disease, Parkinson’s disease

## Abstract

The brain renin-angiotensin system (RAS) has available the necessary functional components to produce the active ligands angiotensins II (AngII), angiotensin III, angiotensins (IV), angiotensin (1–7), and angiotensin (3–7). These ligands interact with several receptor proteins including AT_1_, AT_2_, AT_4_, and Mas distributed within the central and peripheral nervous systems as well as local RASs in several organs. This review first describes the enzymatic pathways in place to synthesize these ligands and the binding characteristics of these angiotensin receptor subtypes. We next discuss current hypotheses to explain the disorders of Alzheimer’s disease (AD) and Parkinson’s disease (PD), as well as research efforts focused on the use of angiotensin converting enzyme (ACE) inhibitors and angiotensin receptor blockers (ARBs), in their treatment. ACE inhibitors and ARBs are showing promise in the treatment of several neurodegenerative pathologies; however, there is a need for the development of analogs capable of penetrating the blood-brain barrier and acting as agonists or antagonists at these receptor sites. AngII and AngIV have been shown to play opposing roles regarding memory acquisition and consolidation in animal models. We discuss the development of efficacious AngIV analogs in the treatment of animal models of AD and PD. These AngIV analogs act via the AT_4_ receptor subtype which may coincide with the hepatocyte growth factor/c-Met receptor system. Finally, future research directions are described concerning new approaches to the treatment of these two neurological diseases.

As life expectancy has increased the incidences of dementia and Parkinson’s disease (PD) have also increased. The number of Alzheimer’s disease (AD) patients in the U.S. is presently estimated to be 4.5 million, with approximately 37 million worldwide (1, 2). By 2040 the worldwide number is predicted to reach 81 million with 4.6 million new patients diagnosed per year (3). There is a 3% occurrence of AD between the ages of 65–74 years, and upwards of 50% for those 85 years of age and older (4). Beyond the cost associated with treatment (estimated range from $70 to 150 billion annually in the U.S. alone) are the personal hardships and sacrifices suffered by family members and other care givers accompanied by the frustrations experienced by the patient and health care professionals as cognitive abilities continue to slowly deteriorate with no efficacious drug treatment available. It is clear that the brain renin-angiotensin system (RAS) is a potential contributor to dementia and blockade of this system has been shown to be important (5–9). However, the precise role(s) played by the brain RAS is unclear and somewhat convoluted given that the octapeptide angiotensin II (AngII) has been shown to disrupt learning and memory; while the hexapeptide angiotensin IV (AngIV) facilitates memory acquisition and consolidation. A second major neurodegenerative disease, PD, was first described by James Parkinson in 1867 and now affects about 10 million people in the U.S. Around the world PD impacts approximately 1% of the population over 50 years of age and 1.5% over 65 years (10). There is accumulating evidence that the brain RAS is important in the etiology of PD as well, and this recently discovered link with the RAS will be discussed.

This review initially describes the presently identified angiotensin ligands and their interaction with specific receptor proteins (AT_1_, AT_2_, and AT_4_). The AT_1_ and AT_2_ receptor subtypes have been well characterized (11, 12); however, the AT_4_ subtype has only been partially sequenced (13). Next we discuss the current hypotheses offered to explain the causes of AD and PD, and the drugs thus far developed to treat these dysfunctions. The role of angiotensins in memory formation and PD is discussed, followed by current attempts to develop new and efficacious treatments for AD and PD. Related to these efforts we describe an AngIV related analog effective in delaying or reversing symptoms in animal models of AD and PD. We conclude with thoughts concerning future directions in these important clinical areas of research.

## Formation of Angiotensin Ligands

**Figure 1:**
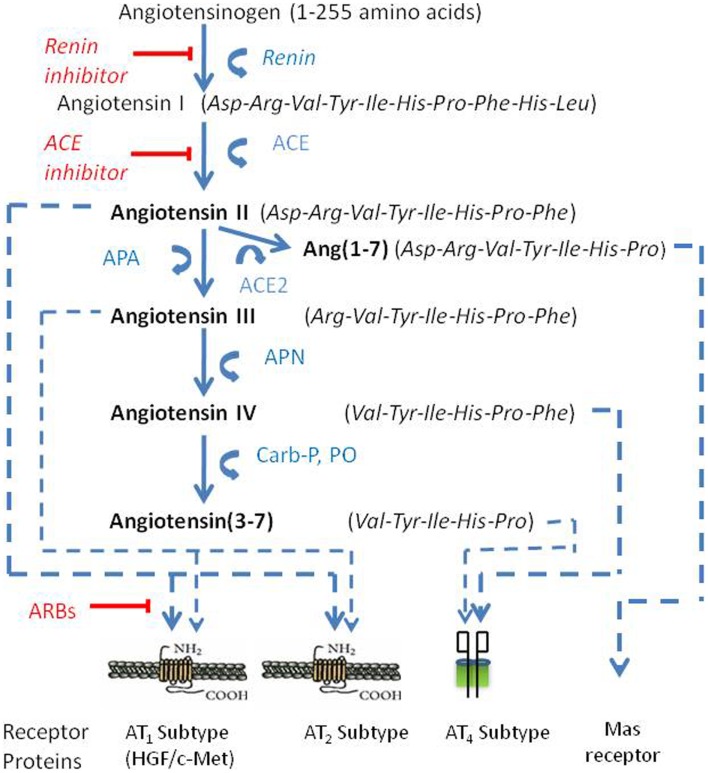
**The renin-angiotensin pathway including active ligands (bold), enzymes, receptors, and inhibitors involved in central angiotensin mediated blood pressure**. Abbreviations: ACE, angiotensin converting enzyme; ACE_2_, angiotensin converting enzyme 2; AP-A, aminopeptidase A; AP-N, aminopeptidase N; ARBs, angiotensin receptor blockers.

Angiotensin peptides are derived from the precursor protein angiotensinogen via several enzymatic conversion pathways [Figure 1; Ref. (14–16)]. Briefly, the decapeptide angiotensin I (AngI) is formed by renin (EC 3.4.23.15) acting upon the amino terminal of angiotensinogen. AngI serves as a substrate for angiotensin converting enzyme (ACE; EC 3.4.15.1) that hydrolyzes the carboxy terminal dipeptide His-Leu to form the octapeptide AngII (14). AngII is converted to the heptapeptide angiotensin III (AngIII) by glutamyl aminopeptidase A (AP-A; EC 3.4.11.7) that cleaves the Asp residue at the N-terminal (17–19). Membrane alanyl aminopeptidase N (AP-N; EC 3.4.11.2) cleaves Arg at the N-terminal of AngIII to form the hexapeptide angiotensin IV (AngIV). AngIV can be further converted to Ang(3–7) by carboxypeptidase P (Carb-P) and prolyl oligopeptidase (PO) cleavage of the Pro-Phe bond to form Ang(3–7).

**Figure 2:**
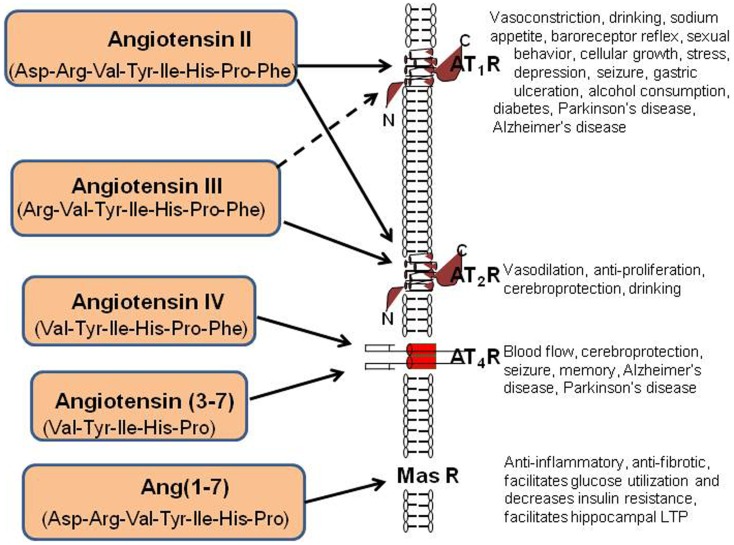
**Description of the peptide structures and enzymes involved in the conversion of angiotensinogen to angiotensin I through shorter angiotensins**. The biologically active forms include angiotensin II, III, IV, angiotensin (3–7), and angiotensin (1–7). The respective receptors where these active angiotensins bind are indicated by arrows.

AngII can also be converted to Ang(1–7) by Carb-P cleavage of Phe (20), by the mono-peptidase ACE_2_ (21), or by ACE cleavage of the dipeptide Phe-His from Ang(1–9) (22). Note that the functional role of insertion of Alu in intron 16 of the human ACE gene has been questioned; however, Wu et al. (23) has shown this form of ACE to upregulate ACE promoter transcriptional activity by approximately 70%. Ang(1–7) is converted to Ang(2–7) by AP-A acting at the Asp-Arg bond (24). AngI is biologically inactive; while AngII and AngIII are full agonists at the AT_1_ and AT_2_ receptor subtypes and mediate pressor and dipsogenic functions [Figure 2; reviewed in Ref. (11)]. AngIV binds with low affinity to the AT_1_ and AT_2_ receptor subtypes (25, 26), but with high affinity and selectivity to the AT_4_ receptor subtype (26–28).

Finally, AngII can be converted to Ang(1–7) by ACE_2_ (29). Recent evidence indicates that this Ang(1–7)/Mas receptor system is important with regard to counteracting peripheral organ inflammation and fibrosis, increasing glucose utilization and decreasing insulin resistance (30, 31). The Mas receptor has been identified in the brain with particularly high concentrations within the dentate gyrus of the hippocampus and piriform cortex (32). In agreement with these memory-related brain distributions of Mas, Ang(1–7) has been shown to facilitate hippocampal long-term potentiation (LTP) (33) suggesting its potential importance in learning and memory. The Ang(1–7)/Mas receptor system also plays a neuroprotective role in responding to cerebral ischemia (34). The reader is referred to the following reviews for detailed characterizations of the angiotensin receptor subtypes (8, 11, 30, 35).

## Current Hypotheses of Alzheimer’s Disease

Two prominent theories are presently offered to explain the neurochemical changes underlying AD. These are the cholinergic and amyloid cascade hypotheses. Based on the cholinergic hypothesis of memory formation it was originally proposed that drugs designed to inhibit central and peripheral acetylcholine esterase (AChE), and serve as a muscarinic M2 autoreceptor antagonist, would result in facilitated release of ACh. Further, AChE binding to the non-amyloidogenic form of β-amyloid peptide (Aβ) appears to facilitate a conformational shift to the amyloidogenic form (36–38). Treatment with an AChE inhibitor would be expected to neutralize the catalytic site of the enzyme and reduce Aβ peptide aggregation as facilitated by active AChE. To date the cholinergic hypothesis of memory formation has driven the development of the major marketed drugs in the form of AChE inhibitors (Tacrine^®^, Donepezil^®^, Rivastigmine^®^, and Galantamine^®^) which will go generic in the near future (9). These drugs are only marginally helpful in treating symptoms and do not appear to impact the underlying neuropathology of this disease (39). The FDA approved Namenda^®^(Memantine HCl) in 2004, an *N*-methyl-d-aspartate (NMDA) receptor antagonist designed to limit glutamate excitotoxicity and intended to treat moderate to severe AD patients (40). Namenda is also limited regarding its ability to slow disease progression and does little to stem the neuropathology. Recent research has focused on the accumulation of brain Aβ as an important target in the pathogenesis of AD (41). There may be a link between Aβ accumulation and NMDA receptor over activation in that oxidative stress, plus the elevated intracellular calcium generated due to Aβ accumulation, appear to enhance glutamate mediated neurotoxicity via increased NMDA receptor activation (42).

There are many possible reasons for the lack of an effective therapy for AD including the complexity of the disease process and the resulting inability to identify reliable biomarkers. In addition, it is now apparent that AD is multifactorial rather than a single disease (43). To further complicate drug development and diagnosis those AD criteria behaviors denoting cognitive decline can also result from a number of other clinical conditions including vascular disease (44, 45), frontotemporal dementia, PD-induced dementia, HIV infection (46, 47), as well as cumulative oxidative damage and toxicities accompanying normal aging (48). The ultimate goal of development must be a drug that prevents the progressive loss of synapses and neurons and reverses this degenerative process.

The second major hypothesis concerns amyloid peptides that range in length from 39 to 42 amino acids and are produced by the conversion of amyloid precursor protein (APP) (49). It is suggested that the cellular accumulation of Aβ(1–42) causes the neurodegenerative characteristics of AD (41). Treatment with the angiotensin receptor blocker (ARB) Valsartan has been shown to discourage amyloid β-mediated cognitive dysfunction in the Tg 2576 mouse model of AD (50). Along these lines, intranasal injection of Losartan (also an ARB) resulted in neuroprotection, presumably via its Aβ-reducing plus anti-inflammatory effects (51).

With the recent clinical trials failure of so called “β-amyloid buster compounds” by Lilly and Pfizer Pharmaceuticals it now appears that both of these hypotheses are much too simple and new approaches must be developed and tested. One very attractive potential upstream contributor to dementia is the brain RAS. A potential role for the brain RAS in learning and memory was proposed some time ago and thus provides justification for the identification of brain RAS components that may serve as targets for the treatment of AD [reviewed in Ref. (52–56)]. Recent findings suggest that many of the memory enhancing effects initially attributed to AngII are likely due to the conversion of AngII to AngIV, and it is this peptide acting as an agonist at the AT_4_ receptor subtype, that is responsible for cognitive facilitation (20, 57, 58). Taken as a whole research findings now suggest that AngII interferes with performance on most memory tasks used with animal models; while AngIV facilitates performance (59). This AngIV memory facilitation hypothesis is consistent with the finding that ARBs improve cognitive processing (60–64). It remains to be determined whether blockade of the AT_1_ receptor subtype permits conversion of excess endogenous AngII to AngIV which then activates the AT_4_ receptor. This notion is also supported by the observation that ACE inhibitors enhance cognitive processing in both humans (65, 66) and animal models (67). Specifically, resulting increases in AngI levels are likely converted to Ang(1–9) and then to AngIII, AngIV, and Ang(3–7). Both AngIV and Ang(3–7) act as agonists at the AT_4_ receptor subtype. See below for further details concerning AngIV-induced memory facilitation. It should be noted that ACE has been shown to convert Aβ1–42 to Aβ1–40 (39). Aβ1–42 is the form that appears to be responsible for brain amyloid deposition (9). Thus, treatment with an ACE inhibitor could, over time, result in greater accumulations of amyloid plaques.

## A Role for Angiotensins in Memory Consolidation

A number of studies indicate that AngIV, and AngIV analogs such as Nle^1^-AngIV, facilitate LTP, learning, and memory consolidation (68–72). Studies using various animal models of dementia to test the influence of Nle^1^-AngIV have demonstrated reversal of deficits initiated by: (1) treatment with scopolamine (73); (2) kainic acid injections into the hippocampus (74); (3) perforant path knife-cuts (72); and (4) ischemia resulting from transient four-vessel occlusion (12). Consistent with these behavioral and electrophysiological results, brain autoradiography-determined binding sites for [^125^I]-AngIV have been localized in structures known to mediate cognitive processing including the neocortex, hippocampus, and basal nucleus of Meynert (26, 56, 75). Denny and colleagues (76) reported that AngII blocked hippocampal LTP *in vivo* in perforant path stimulated dentate gyrus neurons. This inhibition appeared to be dependent upon AngII binding at the AT_1_ receptor subtype given that co-application of Losartan with AngII significantly attenuated this inhibition; while application of the AT_2_ receptor antagonist PD123, 319 failed to interfere with this AngII-induced inhibition (77). Recently it has been established that AngII, chronically perfused via subcutaneous osmotic pump in mice, resulted in hypertension and impaired spatial memory as measured using the Morris water maze task beginning during the third week of treatment (78). Such AngII-induced spatial memory impairment has also been reported in rats following acute intracerebroventricular infusion (79). Significant reductions in cerebral blood flow and brain acetylcholine levels, as well as oxidative stress, were measured 60 min following AngII injection. Taken together these results indicate that AngII generally interferes with learning and memory acquisition.

## Current Hypotheses of Parkinson’s Disease

Parkinson’s disease is due to a progressive loss of dopaminergic (DA) neurons in the substantia nigra *pars compacta*. The striatum is the primary projection field of these substantia nigra neurons, thus the loss of DA results in insufficient stimulation of striatal dopaminergic D_1_ and D_2_ receptors (80, 81). Decreased availability of DA triggers the symptomatic triad of bradykinesia, tremors-at-rest, and rigidity. There is evidence from animal models and PD patients that neuro-inflammatory processes, triggered by reactive oxygen species (ROS), damage mitochondrial membrane permeability, enzymes, and mitochondrial genome resulting in DA cell death (82, 83). l-DOPA is efficacious at controlling motor symptoms in the majority of patients but is ineffective regarding non-motor symptoms. Current treatment strategies to relieve these symptoms include DA replacement via Levodopa (l-DOPA, the precursor of DA), DA receptor agonists, monoamine oxidase B inhibitors, and catechol-*O*-methyltransferase inhibitors, to protect the DA that is formed (84, 85). As the disease progresses periods of decreased mobility, dyskinesia, and spontaneous involuntary movements complicate treatment (86). Thus, in addition to treatment with the DA receptor agonists apomorphine and Levodopa, surgical techniques including pallidotomy and deep brain electrical stimulation may be required (87, 88). Progressive neurodegeneration also impacts additional non-dopaminergic neurotransmitter systems including noradrenergic, cholinergic, and serotonergic (89). As a result, non-motor symptoms may develop including depression, sleep disturbances, dementia, and autonomic nervous system failure (90, 91). l-DOPA is reasonably ineffective at combating non-motor symptoms (90). Current research efforts are three-pronged and directed at extending the duration of Levodopa’s efficacy, controlling these additional non-motor symptoms, and developing new strategies designed to offer neuroprotection and overall disease reversal benefits. Attaining the goal of slowing or reversing the rate of DA neuron loss may also result in the protection of non-DA neurotransmitter systems.

## A Role for Angiotensins in Parkinson’s Disease

Allen et al. (92) were first to suggest a potential relationship between the brain RAS and PD. These investigators measured decreased angiotensin receptor binding in the substantia nigra and striatum in post mortem brains of PD patients. A number of studies support an important role for ACE in this disease. ACE is present in the nigra-striatal pathway and basal ganglia structures (93–95). PD patients treated with the ACE inhibitor perindopril revealed improved motor responses to the DA precursor 3,4-dihydroxy-l-phenylalanine (96). Relative to this treatment with perindopril, elevated striatal DA levels have been measured in mice (97). In addition, ACE has been shown to metabolize bradykinin and thus modulate inflammation, a contributing factor in PD. Activation of the AT_1_ receptor subtype by AngII promotes nicotinamide adenine dinucleotide phosphate (NADPH)-dependent oxidases, a significant source of ROS (98, 99). Treatment with ACE inhibitors has been shown to offer protection against the loss of DA neurons in 1-methyl-4-phenyl-1,2,3,6-tetrahydropyridine (MPTP) animal models (100, 101), as well as the 6-hydroxydopamine (6-OHDA) rat model (102). The likely mechanism underlying this ACE inhibitor-induced protection is a reduction in the synthesis of AngII acting at the AT_1_ receptor subtype [reviewed in Ref. (103)]. It is known that AngII binding at the AT_1_ subtype activates the NADPH oxydase complex, thus providing a major source of ROS (104–106). Further, activation of the AT_1_ receptor results in the stimulation of the NF-kB signal transduction pathway facilitating the synthesis of chemokine, cytokines, and adhesion molecules, all important in the migration of inflammatory cells into regions of tissue injury (107).

If AngII activation of the AT_1_ receptor subtype results in facilitation of the NADPH oxidase complex and formation of free radicals, then blockade of the AT_1_ receptor should serve a protective function. This appears to be the case. Treatment with an AT_1_ receptor blocker (ARB) protects DA neurons in both 6-OHDA (108–110) and MPTP animal models (105, 111, 112). ARBs have been shown to reduce the formation of NADPH oxidase-derived ROS following administration of 6-OHDA (113). While the risk of developing PD is reduced with the use of calcium channel blockers to control hypertension, the positive influences of ACE inhibitors, β-blockers, and ARBs are not clear (114). Of relevance to this issue is the PD patient who showed exacerbated motor dysfunction when treated with an ARB [Losartan; Ref. (115)]. This patient experienced severe bradykinesia while on Losartan, accompanied by frequent episodes of freezing.

The AT_2_ receptor subtype is present in several fetal tissues including uterus, ovary, adrenal gland, heart, vascular endothelium, kidney, and brain (particularly neocortex and hippocampus) (11, 116–119). As the animal matures the expression of the AT_2_ receptor decreases. It appears that adult mammalian brain levels of this receptor in the striatum and substantia nigra are reasonably low (56, 120). The AT_2_ receptor has been linked with cell proliferation, differentiation, and tissue regeneration (121, 122). The results from a study utilizing mesencephalic precursor cells indicated that AngII, acting at the AT_2_ receptor, facilitated differentiation of precursor cells into DA neurons (123). Along these lines, activation of the AT_2_ receptor has been shown to inhibit NADPH oxidase activation (124). However, Rodriguez-Pallares et al. (99) found that AngII treatment of the 6-OHDA lesioned rat increased DA cell death. This could be due to the much greater numbers of brain AT_1_ receptors, as compared with AT_2_ receptors, such that the beneficial effects of AT_2_ receptor activation was overwhelmed by AT_1_ activation. Finally, the expression of AT_2_ receptors in PD patients appears to be decreased in the caudate nucleus but is unchanged in the substantia nigra and putamen (125).

Basal ganglia structures possess a local RAS that evidences increased activity during dopaminergic degeneration (109, 126, 127). Villar-Cheda et al. (128) have reported that reserpine-induced decreases in DA resulted in a significant increase in the expression of AT_1_ and AT_2_ receptors. A similar pattern was seen with 6-OHDA-induced DA denervation in which a decrease in receptor expression was noted with l-DOPA treatment. These results indicate a direct interaction between the RAS and the dopaminergic system in basal ganglia structures. Related to this, Rodriguez-Perez and colleagues (110, 129) used intrastriatal 6-OHDA injections to produce dopaminergic degeneration and noted a significant decrease in DA neurons in ovariectomized rats. This loss of neurons was attenuated by treatment with the AT_1_ receptor antagonist Candesartan, or estrogen replacement. Estrogen replacement also resulted in a down-regulation of AT_1_ receptors and NADPH complex in the substantia nigra, accompanied by an up-regulation of the AT_2_ receptor subtype. These results suggest an important relationship among estrogen levels, brain DA receptors, and the RAS. An increase in the expression of AT_1_ receptors and decreased expression of AT_2_ receptors has been reported in aged rats (130). This observation is of major importance given the potentially deleterious consequences of AT_1_ receptor activation on basal ganglia structures.

Recently Rodriguez-Perez et al. (131) have reported that chronic hypoperfusion in rats resulted in a reduction in striatal DA levels accompanied by a large decline in DA neurons and striatal terminals. This DA neuron loss was countered by orally administered Candesartan. Further, AT_1_ receptor expression was highest in the substantia nigra; while AT_2_ expression was lower in rats that experienced chronic hypoperfusion as compared with controls. Again, Candesartan attenuated such changes in receptor expression. Taken together these findings argue that inhibition of AT_1_ receptor activity serves a neuroprotective role in PD.

The involvement of AngIV in PD has been initially investigated (132). A genetic *in vitro* PD model was used consisting of the α-synuclein over-expression of the human neuroglioma H4 cell line. Results indicated a significant reduction in α-synuclein-induced toxicity with Losartan treatment combined with the AT_2_ receptor antagonist PD123319, in the presence of AngII. Under these same conditions AngIV was only moderately effective. Our laboratory has recently synthesized a metabolically stable AngIV analog that acts by way of the hepatocyte growth factor (HGF)/c-Met receptor system (133–136) to overcome the motor dysfunctions that follow 6-OHDA-induced lesions of the substantia nigra *pars compacta* in the rat (unpublished results). This compound, called Dihexa, significantly improved both rope hang times and stride length over the course of a 48-day treatment period.

Taken together these findings suggest that treatment with an ARB may offer some protection against the risk of developing PD. However, much additional work employing angiotensin mimetics must be completed to better understand the relationship among brain angiotensin receptors, angiotensin ligands, inflammation, and ROS as related to PD.

## AngIV, HGF, and the Brain DA System

Aging is one of the major risk factors predisposing individuals to neurodegenerative diseases (130, 137, 138). The neurodegeneration accompanying aging is dependent in part upon oxidative stress, neuroinflammation, and microglial NADPH oxidase activity. Each is of significant importance regarding DA neuron loss (106, 139). Activation of AT_1_ receptors by AngII has been shown to facilitate DA neuron degeneration by activating microglial NADPH oxidase (109). The activation of AT_1_ receptors by AngII failed to cause DA neuron degeneration when microglial cells were absent (99). Of related importance, Zawada and colleagues (140) recently reported that nigral dopaminergic neurons responded to neurotoxicity-induced superoxide in two waves. First, a spike in mitochondrial hydrogen peroxide was measured 3 h following treatment with an MPTP metabolite (MPP+). Second, by 24 h following treatment hydrogen peroxide levels were further elevated. Treatment with Losartan suppressed this nigral superoxide production suggesting a potentially important role for ARBs in the treatment of PD. Further, AngII binding at the AT_1_ receptor increased DA neuron degeneration initiated by subthreshold doses of DA neurotoxins by stimulating intraneuronal levels of ROS and neuroinflammation by activation of microglial NADPH oxidase (141–144).

From the above observations it follows that AT_1_ receptor blockade should have a neuroprotective effect on DA neurons in PD patients as demonstrated in animal models (112). Less obvious is the likelihood that AT_1_ receptor blockade results in accumulating levels of AngII that are converted to AngIII and then to AngIV. This conversion cascade has been shown to occur intracellularly (145). In fact, this conversion of AngII appears to be necessary for DA release to occur in the striatum (146). Thus, an intriguing alternative explanation of these AT_1_ receptor antagonist results is that the increased endogenous levels of AngIV facilitate activation of the HGF/c-Met receptor system and neuroprotection of DA neurons. In this way AngIV may act in combination with AT_1_ receptor blockade to protect DA neurons. Our laboratory has offered evidence that AngIV, and AngIV analogs, are capable of facilitating HGF/c-Met activity (133). Support for this claim is presented in several recent reports. First we found that the action of AT_4_ receptor antagonists depends on inhibiting the HGF/c-Met receptor system by binding to and blocking HGF dimerization (134, 147). In contrast, AT_4_ receptor agonists facilitate cognitive processing and synaptogenesis by acting as mimics of the dimerization domain of HGF [hinge region; Ref. (135, 148)]. This work has culminated in the synthesis of a small molecule AT_4_ receptor agonist capable of penetrating the blood-brain barrier and facilitating cognitive processing presumably by increasing synaptogenesis (133). This small molecule (MM-201) has a *K*_d_ for HGF ≈6.5 or 13 pM (136). This AngIV-HGF/c-Met interaction could explain earlier reports indicating that activation of the AT_4_ receptor facilitates cerebral blood flow and neuroprotection (149–151).

In agreement with the above findings, HGF has been shown to positively impact ischemic-induced injuries such as cardiac (152) and hind limb ischemia (153, 154). HGF has also been shown to eliminate hippocampal neuronal cell loss in transient global cerebral ischemic gerbils (155), and transient focal ischemic rats (156). Date and colleagues (157, 158) have reported HGF-induced improvements in escape latencies by microsphere embolism-cerebral ischemic rats using a circular water maze task. These authors measured reduced damage to cerebral endothelial cells in ischemic animals treated with HGF. Shimamura et al. (159) have recently shown that over-expression of HGF following permanent middle cerebral artery occlusion resulted in significant recovery of performance in the Morris water maze and passive avoidance conditioning tasks. Treatment with HGF was also found to increase the number of arteries in the neocortex some 50 days following the onset of ischemia.

In sum, these results suggest a role for the HGF/c-Met receptor system in cerebroprotection and are consistent with the notion that AngIV increases blood flow by a NO-dependent mechanism (141). In support of this hypothesis a report by Faure et al. (160) indicated that increasing doses of AngIV via the internal carotid artery significantly decreased mortality and cerebral infarct size in rats 24 h following embolic stroke due to the intracarotid injection of calibrated microspheres. Pretreatment with the AT_4_ receptor antagonist Divalinal-AngIV, or the nitric oxide synthase inhibitor Nω-nitro-l-arginine methyl ester (l-NAME), abolished this protective effect. Sequential cerebral autoradiography indicated that AngIV caused the redistribution of blood flow to ischemic areas within a few minutes. Thus, AngIV may yield its cerebral protective effect against acute cerebral ischemia via an intracerebral-hemodynamic c-Met receptor-mediated NO-dependent mechanism. Should these relationships hold then a metabolically stable blood-brain barrier penetrant small molecule that activates the HGF/c-Met system could prove highly efficacious in the treatment of PD.

## Future Research Directions

The use of ACE inhibitors and AT_1_ and/or AT_2_ receptor blockers have shown preliminary experimental promise in the treatment of stress, depression, alcohol consumption, seizure, AD, PD, and diabetes. A number of AT_1_ receptor antagonists, capable of penetrating the BBB, are now available with new ones in clinical trials (161, 162); however, the vast majority of clinical studies concerned with the use of antihypertensive agents to treat dementia have focused on ACE inhibitors and diuretics (163, 164). This is also true of studies concerned with cerebroprotection against stroke (165). Traditional antidepressant drugs for patients suffering from depression and migraine pathophysiology have taken precedence over the use of ARBs (166). Similarly, the testing of ARBs with seizure and PD patients has yet to gain momentum. The treatment of diabetic patients with ARBs is just now receiving attention (167), particularly with patients suffering diabetic related nephropathy (168, 169). The AngIV/AT_4_ receptor system has been implicated in memory facilitation, cerebroprotection, seizure, Alzheimer’s, and PDs. The lack of BBB penetrating AT_4_ receptor agonists and antagonists has limited our understanding concerning the relative importance of brain AT_1_ and AT_4_ receptor subtypes in the etiology and treatment of dementias, stroke, and related memory dysfunctions. Although current drug development efforts show promise regarding small molecules that interact specifically with the AT_4_ receptor, much additional effort is needed in this important research area.

There remain a number of important unanswered questions regarding whether the observed biological effects of AngIV and its analogs are mediated by the HGF/c-Met system. (1) What is the complete brain distribution of the c-Met receptor and is this receptor expressed in significant levels within cognitive mediating brain structures? (2) Can AngIV, and AngIV analogs, specifically activate the HGF/c-Met receptor system *in vivo* to induce AngIV/AT_4_ receptor associated functions? (3) Are the levels of endogenous AngIV sufficient to augment the HGF-dependent activation of brain c-Met receptors? This is a very significant issue in that the *in vivo* half-life of AngIV appears to be very short. Related to this point, what is the affinity of AngIV for HGF? (4) Does LVV-H7 bind to HGF, and if so, at what affinity? and (5) Does the activation of brain c-Met receptors produce neurogenesis, and if so can this phenomenon be utilized to replace experimentally and clinically damaged pathways? Until these questions are answered an understanding of the true mechanism of action of AngIV and its analogs will remain uncertain.

## Conclusion

The classic RAS was originally described as a circulating hormonal system involved in cardiovascular regulation, vasopressin release, sympathetic activation, and body water/electrolyte balance. These functions appear to be primarily mediated by the AT_1_ receptor subtype. With the recognition that local tissue RASs exist has come research interest in additional physiological and pharmacological functions that permit better understanding of clinical dysfunctions such as inflammation, cellular proliferation, apoptosis, and fibrosis accompanied by an increased appreciation for the role of both the AT_1_ and AT_2_ receptor subtypes [reviewed in Ref. (170, 171)]. It is now clear that the brain RAS is involved in a number of novel physiologies and behaviors that have important implications for the design and development of new drug treatment strategies. This review focused on the importance of the RAS with regard to two neurodegenerative diseases, Alzheimer’s and PDs. The use of ACE inhibitors and ARBs with Alzheimer’s patients suggests an involvement by the brain RAS in this dysfunction. Such positive results force the need to further investigate the potential roles of several angiotensins, not only the AngII/AT_1_ receptor system. Clearly the AngII/AT_2_ receptor and AngIV/AT_4_ (c-Met) receptor systems have been shown to exert positive influences on memory acquisition and retrieval and are worthy of additional attention. The Ang(1–7)/Mas receptor system has been implicated in neuroprotection and the facilitation of LTP and also deserves further experimental evaluation.

Taken together these findings encourage new clinically relevant approaches to understanding the memory enhancing effects, especially of the angiotensin IV system, on cerebral blood flow, neuroprotection, stress and depression, alcohol consumption, seizure, Alzheimer’s and PDs, and diabetes (12, 172, 173). The development of blood-brain barrier permeable AT_4_ receptor agonists and antagonists presents a novel and promising new strategy for the treatment of several of these clinical dysfunctions (174–177).

## Conflict of Interest Statement

The authors declare that the research was conducted in the absence of any commercial or financial relationships that could be construed as a potential conflict of interest.
